# Draft Genome Sequence of *Diplodia seriata* F98.1, a Fungal Species Involved in Grapevine Trunk Diseases

**DOI:** 10.1128/genomeA.00061-17

**Published:** 2017-04-06

**Authors:** Guillaume Robert-Siegwald, Julie Vallet, Eliane Abou-Mansour, Jiabao Xu, Patrice Rey, Christophe Bertsch, Cecilia Rego, Philippe Larignon, Florence Fontaine, Marc-Henri Lebrun

**Affiliations:** a UMR 1290 BIOGER, INRA, AgroParisTech, Campus AgroParistech, Thiverval-Grignon, France; b Université de Reims Champagne-Ardenne, URVVC EA 4707, Laboratoire Stress, Défenses et Reproduction des Plantes, Reims, France; c Plant Biology Department, University of Fribourg, Fribourg, Switzerland; d BGI-Shenzhen, Shenzhen, China; e UMR1065 SAVE, Santé et Agroécologie du Vignoble, INRA, Bordeaux Sciences Agro, Villenave d’Ornon, France; f Université Haute Alsace, Laboratoire Vigne Biotechnologie et Environnement EA 3991, Colmar, France; g Institut Supérieur d’Agronomie, Tapada da Ajuda, Lisbon, Portugal; h Institut Français de la Vigne et du Vin Pôle Rhône-Méditerranée, Rodilhan, France

## Abstract

The ascomycete *Diplodia seriata* is a causal agent of grapevine trunk diseases. Here, we present the draft genome sequence of *D. seriata* isolate F98.1 (37.27 Mb, 512 contigs, 112 scaffolds, and 8,087 predicted protein-coding genes).

## GENOME ANNOUNCEMENT

*Diplodia seriata* (1, 2) is one of the most common species in the family *Botryosphaeriaceae* that is associated with grapevine trunk diseases (3, 4). In grapevine, *D. seriata* is frequently isolated from necrotic tissues (black spots or sectors) localized in the wood of infected trunks or arms (3–5). This species is also frequently isolated from woody tissues of trees such as *Acer* sp., *Prunus* sp., and *Quercus* sp. (2). Pycnidia are produced on infected grapevine wood or pruning shoots, and liberate pycnidiospores dispersed by rainfall or sprinkler irrigation (6, 7). On grapevine surfaces, pycnidiospores germinate and infectious hyphae penetrate into the plant tissues through pruning wounds (4, 5). Inoculation of *D. seriata* on wounded stems from grafted grapevines of the highly susceptible cultivar tempranillo induces a local brown necrosis in all plants and foliar symptoms in 50% of plants (8). *D. seriata* is known to secrete several plant polymers degrading enzymes such as cellulases, xylanases, lipases, and laccases (9, 10). This fungal species also produces phytotoxic secondary metabolites such as (−)-mellein and its derivates (11, 12).

*D. seriata* F98.1 was isolated in 1998 at Perpignan, France, from the trunk of a grapevine exhibiting foliar symptoms typical of Syrah decline (13). *D. seriata* F98.1 is pathogenic on grapevine (8, 13). Sequencing was performed by BGI-Tech (China) using Illumina HiSeq 2500 at a coverage of 270× (170-, 500-, and 6,000-bp libraries). After quality filtering, a total of 88,596,792 paired-end reads of 125 bp were obtained. Assembly with SOAPdenovo version 1.05 (14) led to 512 contigs and 112 scaffolds (37.27 Mb; G+C%: 56.8). This high-quality genome (scaffold *N*_50_: 2.9 Mb; minimum scaffold length: 1,007 bp; gaps: 250 kb) has 13 scaffolds with a size greater than 1 Mb (90% of the total sequence), likely corresponding to chromosomes. Using GLEAN (15), 8,087 coding sequences (CDSs) were identified, 93% being supported by RNAseq (mycelium on potato dextrose broth or minimal medium for 4 days). Recently, the genome sequence of *D. seriata* DS831, isolated from an infected grapevine (United States, 2011), was released (16). The genome size of DS831 (37.13 Mb) is similar to the genome size of F98.1, but its assembly is five times more fragmented (1,391 contigs; 695 scaffolds), and it carries 9,398 CDSs. Bidirectional best BLAST hit (BDBH) analysis revealed that 82% of F98.1’s and DS831’s CDSs are similar. OrthoFinder (17) identified 5,935 orthologous single-copy gene families shared between the two genomes. According to BDBH analysis, 1,507 genes are specific to strain F98.1, and 2,763 are specific to strain DS831. Using BLASTN, we found that 2,686 (97.2%) of the genes thought to be specific to strain DS831 were present in the F98.1 genome sequence, and 1,440 (95.6%) of the genes thought to be specific to strain F98.1 are present in the DS831 genome sequence. The difference in CDS numbers between DS831 and F98.1 is likely a consequence of using different annotation software (GLEAN versus Augustus). Fusing the two annotations produces a set of 10,773 CDSs (8,087 from F98.1 and 2,686 from DS831).

### Accession number(s).

This whole-genome project has been deposited at NCBI GenBank under the accession number MSZU00000000. The version described in this paper is the first version, MSZU01000000.

## References

[1] Phillips AJL, Crous PW, Alves A 2007 *Diplodia seriata*, the anamorph of “*Botryosphaeria*” *obtusa*. Fungal Divers 25:141–155.

[2] Phillips AJL, Alves A, Abdollahzadeh J, Slippers B, Wingfield MJ, Groenewald JZ, Crous PW 2013 The *Botryosphaeriaceae*: genera and species known from culture. Stud Mycol 76:51–167. doi:10.3114/sim0021. 24302790 PMC3825232

[3] Urbez-Torres JR 2011 The status of *Botryosphaeriaceae* species infecting grapevines. Phytopathologia Mediterr 50:5–45.

[4] Bertsch C, Ramírez-Suero M, Magnin-Robert M, Larignon P, Chong J, Abou-Mansour E, Spagnolo A, Clément C, Fontaine F 2013 Grapevine trunk diseases: complex and still poorly understood. Plant Pathol 62:243–265. doi:10.1111/j.1365-3059.2012.02674.x.

[5] Larignon P 2011 Les maladies du bois de la vigne. Quelques éléments sur la biologie de deux champignons associés, *Phaeoacremonium aleophilum* & *Diplodia seriata*. Phytoma 646:41–44.

[6] Kuntzmann P, Villaume S, Bertsch C 2009 Conidia dispersal of *Diplodia* species in a French vineyard. Phytopathologia Mediterr 48:150–154.

[7] Úrbez-Torres JR, Peduto F, Gubler WD 2010 First report of grapevine cankers caused by *Lasiodiplodia crassispora* and *Neofusicoccum mediterraneum* in California. Plant Dis 94:785–785. doi:10.1094/PDIS-94-6-0785B. 30754340

[8] Reis P, Magnin-Robert M, Nascimento T, Spagnolo A, Abou-Mansour E, Fioretti C, Clément C, Rego C, Fontaine F 2016 Reproducing *Botryosphaeria* dieback foliar symptoms in a simple model system. Plant Dis 100:1071–1079. doi:10.1094/PDIS-10-15-1194-RE. 30682279

[9] Bénard-Gellon M, Farine S, Goddard ML, Schmitt M, Stempien E, Pensec F, Laloue H, Mazet-Kieffer F, Fontaine F, Larignon P, Chong J, Tarnus C, Bertsch C 2015 Toxicity of extracellular proteins from *Diplodia seriata* and *Neofusicoccum parvum* involved in grapevine Botryosphaeria dieback. Protoplasma 252:679–687. doi:10.1007/s00709-014-0716-y. 25323623

[10] Esteves AC, Saraiva M, Correia A, Alves A 2014 Botryosphaeriales fungi produce extracellular enzymes with biotechnological potential. Can J Microbiol 60:332–342. doi:10.1139/cjm-2014-0134. 24802941

[11] Djoukeng JD, Polli S, Larignon P, Abou-Mansour E 2009 Identification of phytotoxins from *Botryosphaeria obtusa*, a pathogen of black dead arm disease of grapevine. Eur J Plant Pathol 124:303–308. doi:10.1007/s10658-008-9419-6.

[12] Andolfi A, Mugnai L, Luque J, Surico G, Cimmino A, Evidente A 2011 Phytotoxins produced by fungi associated with grapevine trunk diseases. Toxins (Basel) 3:1569–1605. doi:10.3390/toxins3121569. 22295177 PMC3268457

[13] Larignon P, Fulchic R, Cere L, Dubos B 2001 Observation on black dead arm in French vineyards. Phytopathologia Mediterr 40:336–342.

[14] Luo R, Liu B, Xie Y, Li Z, Huang W, Yuan J, He G, Chen Y, Pan Q, Liu Y, Tang J, Wu G, Zhang H, Shi Y, Liu Y, Yu C, Wang B, Lu Y, Han C, Cheung DW, Yiu SM, Peng S, Xiaoqian Z, Liu G, Liao X, Li Y, Yang H, Wang J, Lam TW, Wang J 2012 SOAPdenovo2: an empirically improved memory-efficient short-read de novo assembler. Gigascience 1:18. doi:10.1186/2047-217X-1-18. 23587118 PMC3626529

[15] Elsik CG, Mackey AJ, Reese JT, Milshina NV, Roos DS, Weinstock GM 2007 Creating a honey bee consensus gene set. Genome Biol 8:R13. doi:10.1186/gb-2007-8-1-r13. 17241472 PMC1839126

[16] Morales-Cruz A, Amrine KC, Blanco-Ulate B, Lawrence DP, Travadon R, Rolshausen PE, Baumgartner K, Cantu D 2015 Distinctive expansion of gene families associated with plant cell wall degradation, secondary metabolism, and nutrient uptake in the genomes of grapevine trunk pathogens. BMC Genomics 16:469. doi:10.1186/s12864-015-1624-z. 26084502 PMC4472170

[17] Emms DM, Kelly S 2015 OrthoFinder: solving fundamental biases in whole genome comparisons dramatically improves orthogroup inference accuracy. Genome Biol 16:157. doi:10.1186/s13059-015-0721-2. 26243257 PMC4531804

